# High-Dose Ipilimumab and High-Dose Interleukin-2 for Patients With Advanced Melanoma

**DOI:** 10.3389/fonc.2019.01483

**Published:** 2020-01-10

**Authors:** Ann W. Silk, Howard L. Kaufman, Brendan Curti, Janice M. Mehnert, Kim Margolin, David McDermott, Joseph Clark, Jenna Newman, Praveen K. Bommareddy, Lisa Denzin, Saltanat Najmi, Azra Haider, Weichung Shih, Michael P. Kane, Andrew Zloza

**Affiliations:** ^1^Department of Medical Oncology, Dana-Farber Cancer Institute and Department of Medicine, Harvard Medical School, Boston, MA, United States; ^2^Robert Wood Johnson Medical School, Rutgers Cancer Institute of New Jersey, New Brunswick, NJ, United States; ^3^Replimune, Woburn, MA, United States; ^4^Massachusetts General Hospital, Boston, MA, United States; ^5^Earle a Chiles Research Institute, Providence Cancer Institute, Portland, OR, United States; ^6^City of Hope, Duarte, CA, United States; ^7^Beth Israel Deaconess Medical Center and Department of Medicine, Harvard Medical School, Boston, MA, United States; ^8^Loyola University Medical Center, Maywood, IL, United States; ^9^Biomedical Health Sciences, Rutgers University, New Brunswick, NJ, United States; ^10^Department of Pediatrics, Child Health Institute of New Jersey, Robert Wood Johnson Medical School, New Brunswick, NJ, United States; ^11^Amgen, Thousand Oaks, CA, United States; ^12^Bristol Myers-Squibb, Princeton, NJ, United States; ^13^Department of Internal Medicine, Rush University Medical Center, Chicago, IL, United States

**Keywords:** melanoma, interleukin-2, cytokine, ipilimumab, combination immunotherapy, hepatitis, antigen presentation

## Abstract

High-dose ipilimumab (IPI) and high-dose interleukin-2 (IL-2) are approved agents for metastatic melanoma, but the efficacy and safety of the combination are unknown. The objective of this study was to evaluate the feasibility, safety, and efficacy of combination high-dose IPI and high-dose IL-2 in patients with histologically confirmed advanced unresectable stage III and IV melanoma. This Phase II, multicenter, open-label, single-arm trial was conducted in nine patients enrolled between 12/2014 and 12/2015. Subjects were treated with high-dose IPI 10 mg/kg intravenous (IV) every 3 weeks for four doses starting at week 1 and high-dose IL-2 (600,000 IU/kg IV bolus every 8 h for up to 14 doses) concurrently with IPI at weeks 4 and 7. After the first 12 weeks of combination therapy, maintenance IPI (10 mg/kg IV) monotherapy was administered every 12 weeks for up to 1 year. No patient had received prior PD-1 blockade, and only one received prior vemurafenib. Confirmed partial response was achieved in one (11%), stable disease in four (44%), and progressive disease in four (44%) of nine patients. Two patients achieved durable disease control of 44+ and 50+ months at the most recent follow-up without subsequent therapy. The median overall survival was not reached after a minimum 24 months of follow-up time. One-year and 2-year survival rates were 89 and 67%, respectively. Seven patients (78%) experienced grade 3 or 4 adverse events related to the study therapy, three of which were attributed to both agents. One patient discontinued the treatment due to liver and kidney toxicity. While toxicity was significant, all events were reversible, and there was no treatment-related mortality. In peripheral blood of patients with decreasing tumor burden, the ratio of the non-classical MHC-II proteins HLA-DM to HLA-DO increased 2-fold, raising the possibility of the ratio of HLA-DM:HLA-DO as a novel biomarker of response to treatment. Although the sample size was limited, combination therapy with high-dose IPI and high-dose IL-2 was feasible and associated with clinical benefit. IL-2-based compounds in combination with CTLA-4 blockade should be studied in advanced melanoma patients who fail to benefit from first-line PD-1 blockade.

**Clinical Trial Registration:** ClinicalTrials.gov, NCT02203604. Registered 30 July 2014, https://clinicaltrials.gov/ct2/show/NCT02203604.

## Introduction

The cytotoxic T lymphocyte antigen 4 (CTLA-4) checkpoint inhibitor, ipilimumab (IPI), which sustains T cell activity by blocking T cell response inhibition, is associated with a 15–20% response rate in melanoma patients (1) and with long-term progression-free survival of 21% (2). High-dose interleukin-2 (IL-2) administered as first-line therapy for patients with advanced melanoma results in a response rate of 15–20%, of which approximately one-third are durable complete responses (CRs) sometimes lasting for decades (3). High-grade but short-lived toxicities necessitate inpatient administration and have limited the widespread use of IL-2, but it can be delivered safely in experienced centers with appropriate supportive care (4–6). The role of high-dose IL-2 in subsequent lines of therapy for patients treated with first-line PD-1 antibody (pembrolizumab or nivolumab) is less clear, but retrospective, pooled datasets have suggested similar activity for high-dose IL-2 in this setting (7). CTLA-4 is expressed after T cell activation, and blocking this inhibitory signal while driving T cell proliferation, differentiation, and cytotoxicity with IL-2 is a rational treatment strategy. In fact, a phase I/II trial of IPI followed by IL-2 has previously been conducted, but with IPI doses only up to 2 mg/kg (8, 9). Responses were observed in eight of 36 (22%) patients (including 6 CRs). Five of 36 (14%) patients developed grade 3–4 IPI-related toxicities, but all were reversible. High-dose IPI (10 mg/kg) is significantly more effective than 3 mg/kg (HR for OS = 0.84, 95% CI 0.70–0.99, *p* = 0.04), but it is also more toxic, with grade 3–4 diarrhea in 10% and colitis in 5% of patients (10). Therefore, we conducted a trial to determine the feasibility, efficacy, and safety of combination high-dose IPI and high-dose IL-2 in patients with metastatic melanoma. We chose the sequence of IPI followed by IL-2 based on our hypothesis that IPI could prevent T cell exhaustion induced by IL-2-driven T cell proliferation.

## Methods

### Patient Selection

Adults with histologically confirmed unresectable stage III and IV melanoma and ECOG performance status 0–1 were enrolled at Rutgers Cancer Institute of New Jersey and Providence Cancer Institute. Main exclusion criteria were primary ocular, active brain metastases, active autoimmune disease, concurrent systemic immunosuppressive therapy, significant cardiopulmonary disease, and organ dysfunction. Patients with prior treatment with IPI or IL-2 were excluded. Prior PD-1-directed therapy and BRAF-directed therapy was allowed.

### Design

This was a single-arm study with a primary endpoint of objective response rate in the first 24 weeks of treatment, reported with a 95% confidence interval (CI). The protocol (CINJ#091309) was approved by institutional IRBs and registered (NCT02203604). All patients gave written informed consent. Secondary endpoints included safety, feasibility, overall survival, 1- and 2-year survival, progression-free survival, and best overall response. The planned target sample size was up to 82 patients, but the sponsor stopped the trial early due to slow enrollment.

### Treatment

All patients received induction with IPI (10 mg/kg IV every 3 weeks for four doses) starting at Week 1. At weeks 4 and 7, patients also received high-dose IL-2 (600,000 IU/kg IV bolus every 8 h for up to 14 doses, as tolerated) immediately following IPI. IL-2 dose was calculated using actual body weight, although adjustment to ideal body weight for obese patients was allowed. Following IPI induction, maintenance IPI (10 mg/kg IV) was administered every 12 weeks for four doses. Dose reductions were not permitted for either drug. Both drugs were held and/or discontinued for severe autoimmune toxicity. A physical examination and laboratory tests (including CBC with differential and comprehensive metabolic profile, including liver function and thyroid tests) were done at screening and every 3 weeks. Safety assessments were performed daily during hospitalization for IL-2 therapy. Imaging for tumor assessments was performed every 12 weeks. Response was assessed using WHO criteria modified for immune-related response (11). Adverse events (AEs) were evaluated and graded using NCI Common Toxicity Criteria v4.0.

### Immune Studies and Statistical Analysis

Blood was collected at weeks 1, 4, 7, 12, and 24. Serum was analyzed for cytokines using the LEGENDPlex human CD8/NK panel (BioLegend), and peripheral blood mononuclear cells (PBMCs) were analyzed using flow cytometry. For intracellular measurement of HLA-DM and HLA-DO levels, samples were incubated with antibodies to identify peripheral B cells (CD45+ CD19+ MHC-II+), fixed and permeabilized with Cytofix/Cytoperm (BD PharMingen), and stained with antibodies specific for HLA-D (12) and HLA-DO (13). Ratios of the non-classical MHC-II proteins, HLA-DM, and HLA-DO, are a novel marker of antigen presentation potential and was obtained by dividing the mean fluorescent intensity levels obtained for HLA-DM by that obtained for HLA-DO (14, 15). Levels were compared in patients with decreasing vs. increasing tumor burden using two-way ANOVA with Bonferroni correction for multiple comparisons. Descriptive statistics were used for clinical outcome and safety reporting.

## Results

### Patient Characteristics and Treatment Delivery

**Figure 1:**
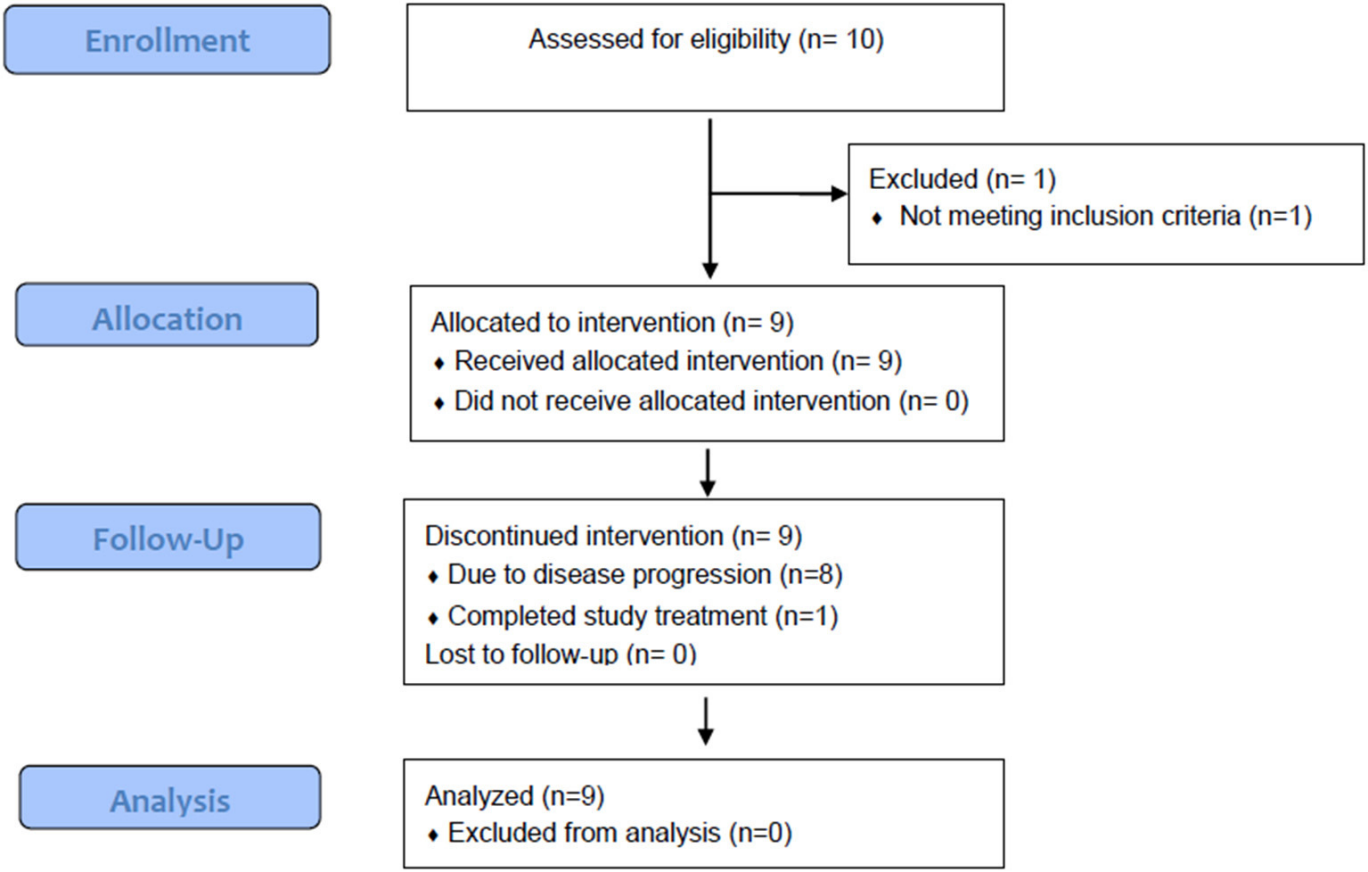
CONSORT flow diagram demonstrates the flow of subjects throughout the study.

Although the trial was stopped in May 2016 due to discontinuation of funding, the safety and efficacy of treatment for nine patients enrolled between December 2014 and December 2015 are reported (Figure 1). Baseline patient characteristics are summarized in Table 1. Four patients with cutaneous, four with acral, and one with anal mucosal primary melanoma were enrolled. All patients had an ECOG performance status of 0. No patients received anti-PD-1 prior to enrolling. Two patients had received adjuvant interferon, and one received vemurafenib in the metastatic setting. Patients received a median of four IPI (range, 2–7) and 11 IL-2 (range, 3–18) doses. No patients received more than two cycles (one course) of IL-2.

**Table 1:** Baseline characteristics of patients treated.

**Age (median years and range)**	**52.5 (25.0–65.6)**
**Gender**	
Male	5 (56%)
Female	4 (44%)
**Race**	
Caucasian	9 (100%)
**Ethnicity**	
Non-Hispanic	6 (67%)
Hispanic	3 (33%)
**ECOG PS**	
0	9 (100%)
**Prior treatment**	
Any	3 (33%)
Adjuvant interferon	2 (22%)
Targeted therapy (vemurafenib)	1 (11%)
**Stage (AJCC 7th)**	
IIIC, unresectable	1 (11%)
IV	8 (89%)
**Primary site**	
Cutaneous	4 (44%)
Acral	4 (44%)
Mucosal	1 (11%)

### Safety

There were no treatment-related deaths. Seven of nine patients (78%) experienced a grade 3 or 4 treatment-related adverse event (AE), three of which were attributed to both agents (Table 2). The most frequent grade 3 and 4 AEs were liver and kidney injury, which occurred during the first 12 weeks of therapy in four patients. One patient experienced grade 4 hyperbilirubinemia, grade 3 transaminase elevation, and grade 4 acute kidney injury during the first cycle of combination high-dose IL-2 and high-dose IPI. The patient was given corticosteroids, and liver and kidney injury resolved 1 month later. The patient was removed from the study due to unacceptable toxicity. Another patient experienced grade 3 transaminase elevation, hyponatremia, and hypotension related to both agents. Approximately 2 weeks after discontinuation of high-dose IL-2, the transaminases rose to grade 4 and the patient required corticosteroid and mycophenolate mofetil for 2 months before the hepatitis resolved. A third patient had grade 4 hematologic toxicity, grade 3 anemia, grade 4 thrombocytopenia, and grade 3 acute kidney injury after the second cycle of combination high-dose IL-2 and high-dose IPI. A bone marrow biopsy showed adequate megakaryocytes, and the patient was treated with steroids for autoimmune thrombocytopenia for ~1 month with eventual resolution of platelet count. A fourth patient had grade 4 transaminase elevation during the first 12 weeks that was attributed only to IPI. This patient improved without steroids and went on to complete 1 year of study therapy with maintenance IPI. Colitis and serious diarrhea were not observed. Grade 1 and 2 diarrhea was noted in four and one patients, respectively.

**Table 2:** Frequency of treatment-related adverse events (AEs).

**System**	**All grades**	**Grade 1**	**Grade 2**	**Grade 3**	**Grade 4**
**(A)**					
Cardiovascular	5 (56%)	2 (22%)	3 (33%)	4 (44%)	0 (0%)
Eye	0 (0%)	0 (0%)	0 (0%)	0 (0%)	0 (0%)
Gastrointestinal	8 (89%)	7 (78%)	4 (44%)	3 (33%)	1 (11%)
General/constitutional	6 (67%)	6 (67%)	2 (22%)	1 (11%)	0 (0%)
Hematologic	2 (22%)	2 (22%)	1 (11%)	1 (11%)	1 (11%)
Infectious	0 (0%)	0 (0%)	0 (0%)	0 (0%)	0 (0%)
Metabolic/nutritional	0 (0%)	0 (0%)	0 (0%)	0 (0%)	0 (0%)
Musculoskeletal	0 (0%)	0 (0%)	0 (0%)	0 (0%)	0 (0%)
Psychiatric	0 (0%)	0 (0%)	0 (0%)	0 (0%)	0 (0%)
Pulmonary	0 (0%)	0 (0%)	0 (0%)	0 (0%)	0 (0%)
Renal/electrolyte	7 (78%)	3 (33%)	2 (22%)	6 (67%)	1 (11%)
Skin	5 (56%)	4 (44%)	3 (33%)	0 (0%)	0 (0%)
Any	9 (100%)	8 (89%)	8 (89%)	7 (78%)	2 (22%)
**(B)**					
Cardiovascular	1 (11%)	0 (0%)	1 (11%)	1 (11%)	0 (0%)
Eye	1 (11%)	0 (0%)	1 (11%)	0 (0%)	0 (0%)
Gastrointestinal	7 (78%)	6 (67%)	4 (44%)	3 (33%)	3 (33%)
General/constitutional	5 (56%)	3 (33%)	3 (33%)	0 (0%)	0 (0%)
Hematologic	2 (22%)	2 (22%)	2 (22%)	1 (11%)	1 (11%)
Infectious	0 (0%)	0 (0%)	0 (0%)	0 (0%)	0 (0%)
Metabolic/nutritional	0 (0%)	0 (0%)	0 (0%)	0 (0%)	0 (0%)
Musculoskeletal	1 (11%)	0 (0%)	1 (11%)	0 (0%)	0 (0%)
Psychiatric	0 (0%)	0 (0%)	0 (0%)	0 (0%)	0 (0%)
Pulmonary	1 (11%)	1 (11%)	0 (0%)	0 (0%)	0 (0%)
Renal/electrolyte	3 (33%)	1 (11%)	1 (11%)	3 (33%)	1 (11%)
Skin	6 (66%)	5 (56%)	4 (44%)	0 (0%)	0 (0%)
Any	9 (100%)	8 (89%)	6 (67%)	4 (44%)	4 (44%)
**(C)**					
Cardiovascular	1 (11%)	0 (0%)	1 (11%)	1 (11%)	0 (0%)
Eye	0 (0%)	0 (0%)	0 (0%)	0 (0%)	0 (0%)
Gastrointestinal	5 (56%)	4 (44%)	2 (22%)	2 (22%)	1 (11%)
General/constitutional	4 (44%)	3 (33%)	2 (22%)	0 (0%)	0 (0%)
Hematologic	1 (11%)	1 (11%)	1 (11%)	1 (11%)	1 (11%)
Infectious	0 (0%)	0 (0%)	0 (0%)	0 (0%)	0 (0%)
Metabolic/nutritional	0 (0%)	0 (0%)	0 (0%)	0 (0%)	0 (0%)
Musculoskeletal	0 (0%)	0 (0%)	0 (0%)	0 (0%)	0 (0%)
Psychiatric	0 (0%)	0 (0%)	0 (0%)	0 (0%)	0 (0%)
Pulmonary	0 (0%)	0 (0%)	0 (0%)	0 (0%)	0 (0%)
Renal/electrolyte	3 (33%)	1 (11%)	1 (11%)	3 (33%)	1 (11%)
Skin	4 (44%)	3 (33%)	3 (33%)	0 (0%)	0 (0%)
Any	7 (78%)	7 (78%)	5 (56%)	3 (33%)	2 (22%)

*Number of subjects (%) with AEs related to **(A)** high-dose IL-2, **(B)** high-dose IPI, or **(C)** combination high-dose IPI and high-dose IL-2*.

### Efficacy

**Figure 2:**
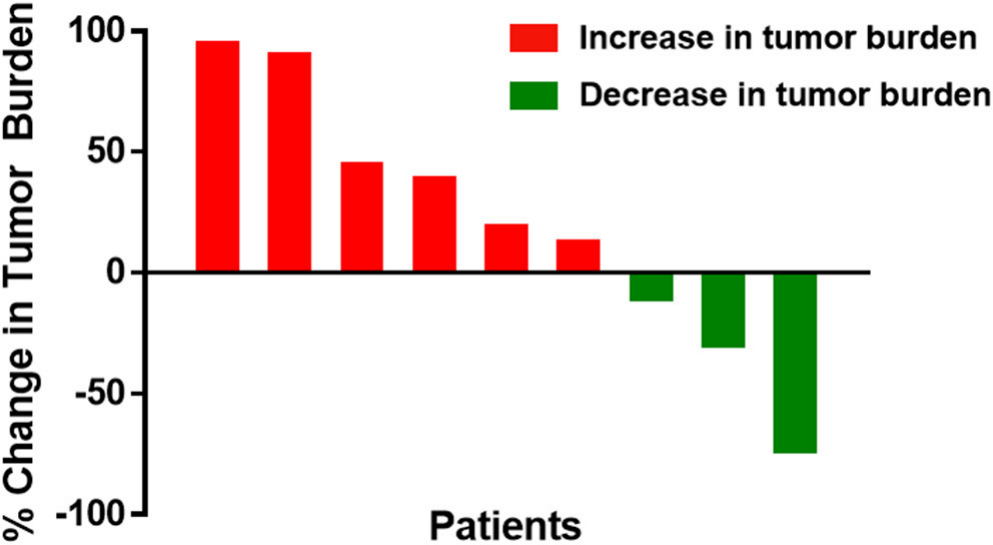
Waterfall plot of best overall response by week 24 for individual metastatic melanoma patients treated with high-dose IPI and high-dose IL-2. Bars represent the best overall response by week 24 as a percent change in tumor burden for each treated patient. Red bars denote patents with increased tumor burden, and green bars denote patients with decreased tumor burden.

Best overall response by week 24 was partial response in one patient (11%; 95% CI 0.57–43.5%) and stable disease in four patients (44%), with progressive disease in four patients (44%; Figure 2). Minimum follow-up time was 24 months. The median OS was not reached, with 1- and 2-year OS rates of 89 and 67%, respectively. Six patients received post-study therapy for progressive disease, most commonly PD-1-directed checkpoint blockade. The patient with the partial response was a 53-year-old woman with a cutaneous primary melanoma of the lower extremity, who experienced an 74% reduction in tumor burden at week 24. Her response was ongoing at 50.0 months of follow-up without subsequent therapy. A patient with stable disease had clear clinical benefit not meeting response criteria. This patient was a 50-year-old man with acral melanoma of the toe web, who experienced a 30% reduction in tumor burden at week 24 and continues without progression at 44.2 months of follow-up.

### Immune Responses

**Figure 3:**
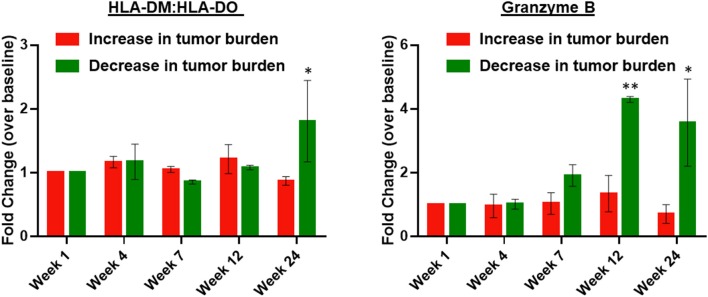
Peripheral blood HLA-DM:HLA-DO ratio and serum granzyme B are elevated in patients with decreased tumor burden following treatment with high-dose IPI and high-dose IL-2. The left panel shows the fold-change in HLA-DM:HLA-DO expression measured by flow cytometry within peripheral blood B cells (CD45+ CD19+ MHC-II+) of patients with an increase in tumor burden (*n* = 6) vs. patients with a decrease in tumor burden (*n* = 3). The right panel shows the fold-change in serum granzyme B over baseline (week 1) in patients with an increase in tumor burden (*n* = 6) vs. patients with a decrease in tumor burden (*n* = 3). Two-way ANOVA with Bonferroni correction; *P < 0.05 and **P < 0.01. Error bars represent S.E.M.

Patients were divided based on response, specifically increased vs. decreased tumor burden. Granzyme B, a marker of cytolytic potential, and HLA-DM:HLA-DO ratio, a marker of antigen presentation capability (14, 15), were significantly increased in patients with decreased tumor burden at weeks 12–24 (Figure 3). No differences were observed in CD8+ effector or CD4+ regulatory T cells (data not shown).

## Discussion

The therapeutic landscape for melanoma has changed dramatically over the past decade, with 11 new agents approved since 2011 (16). This inlcudes multiple small molecule inhibitors of the MAPK pathway and three immune checkpoint inhibtiors. Yet, many patients do not respond to treatment or develop secondary resistance after exposure to individual agents and/or regimens. The potential to improve responses by combination treatment with two agents has emerged as a major area of clinical investigation, and has been largely supported by an improvement in clinical outcomes for combination IPI and nivolumab, although this regimen has been associated with significant toxicity (17). Since combination of approved melanoma agents is now a high priority, we sought to determine the feasibility, safety, and initial response rate of high-dose IPI and high-dose IL-2.

Combination high-dose IPI and high-dose IL-2 treatment was associated with significant AEs, including grade 3–4 AEs in seven of nine patients (78%). There were no new safety signals observed; however, expected IL-2-related side effects such as liver and kidney injury were more prolonged than usual, and systemic corticosteroids were required to treat immune related adverse events in three patients. There was no mortality and surprisingly, no serious diarrhea or colitis was observed. We did confirm the feasibility of combination high-doses IPI and high-dose IL-2, but our results are limited by the small sample size due to loss of funding and premature closure of the study. Of the nine patients who received treatment, there was only 1 responder. Two patients were alive without disease progression after 44+ and 50+ months without subsequent therapy. These durable responses consistent with previous experience with high-dose IL-2 therapy. These data suggest that the combination was tolerated, and further investigation is warranted to better define the response rate of this combination, but not with high dose IPI. Notably, in the previous study by Maker et al. (8), the response rate was higher, despite using very low doses of IPI, suggesting that high dose IPI (10 mg/kg) does not improve outcomes with IL-2 and should not be tested further.

Our data suggest that response to combination high-dose IPI and high-dose IL-2 is associated with markers of antigen presenting machinery regulated by the ratio of non-classical MHC-II proteins HLA-DM and HLA-DO. HLA-DM catalyzes the peptide loading of MHC-II molecules. HLA-DM is negatively regulated by HLA-DO; thus, a high HLA-DM:HLA-DO indicates that the antigen presentation machinery is optimally poised for peptide loading. Serial sampling of peripheral blood in patients treated with combination high-dose IPI and high-dose IL-2 identified that patients with decreased tumor burden had a higher ratio of the non-classical MHC-II HLA-DM:HLA-DO protein levels. HLA-DM promotes peptide loading into the MHC-II pocket by catalyzing the removal of CLIP, and HLA-DM is negatively regulated by HLA-DO. Thus, a high HLA-DM:HLA-DO ratio indicates that the antigen presentation machinery is poised for peptide loading (14, 15). The importance of MHC proteins as predictve biomarkers is demonstrated in a previous report in which MHC-I expression (although not MHC-II expression) was show to be associated with favorable response to ipilimumab (18); thus, our findings support the notions that effective antigen presentation is a key component of the mechanism of action of checkpoint blockade, and MHC protiens are emerging as predictive biomarkers. In addition, we observed that activated lymphocyes with increased cytolytic potential as demonstrated by enhanced granzyme B expression were associated with favorable response.

Our experience highlights potential opporunites and challenges in conducting such clinical trials in the contemporary period. According to a prospectively-collected registry study, high-dose IL-2 retains its efficacy in the post-anti-PD-1 monotherapy setting (7); therefore, safety and activity with this combination in the setting of disease progression following anti-PD-1 treatment may inform the development of novel formulations of IL-2 as salvage therapies. In particular, the use of modified IL-2 molecules designed to promote effector CD8+ T cell responses and limit CD4+ regulatory T cell responses may offer a better strategy for IL-2-based combinations (19). Future studies of IPI and IL-2 should utilize low doses of IPI and most likely will incorporate novel IL-2-based compounds.

## Data Availability Statement

The datasets generated for this study are available on request to the corresponding author.

## Ethics Statement

The studies involving human participants were reviewed and approved by Rutgers University. The patients/participants provided their written informed consent to participate in this study.

## Author Contributions

AS, HK, BC, JM, KM, DM, JC, WS, MK, and AZ contributed to the study concept, design, analysis and interpretation of data, and drafting of the manuscript. AS, HK, BC, JN, PB, LD, SN, AH, WS, and AZ analyzed and interpreted data, and helped draft the manuscript. AS, HK, BC, SN, AH, and AZ were involved in data acquisition. All authors contributed to the revisions of and approved the final manuscript.

### Conflict of Interest

AS reports receiving research funding from Merck and consulting fees from Bristol Myers-Squibb and Merck. HK and PB are employees of Replimune, Inc. DM reports receiving research funding from Prometheus Laboratories and Bristol Myers-Squibb, and consulting fees from Bristol Myers-Squibb, Pfizer, Merck, Novartis, Array BioPharma, Eli Lilly, EMD Serono, Jounce Therapeutics, Peloton, and Alkermes. JC reports receiving research funding from Bristol Myers-Squibb and from Prometheus, an unpaid advisory role for Prometheus, and speaking fees from Bristol Myers-Squibb. LD reports receiving research funding from Janssen. SN is an employee of Amgen. AH is an employee of Docs Global CRO and a consultant for Bristol-Myers Squibb. AZ reports receiving research funding from Merck and Fox Chase Chemical Diversity Center. The remaining authors declare that the research was conducted in the absence of any commercial or financial relationships that could be construed as a potential conflict of interest. The reviewer KZ declared a shared affiliation, with no collaboration, with the author KM to the handling editor at the time of review.

## Abbreviations

IPI: ipilimumab
IL-2: interleukin-2
IV: intravenous
CTLA-4: cytotoxic T lymphocyte antigen 4
CR: complete response
PBMC: peripheral blood mononuclear cell.
